# Chronic hypoxia remodels the tumor microenvironment to support glioma stem cell growth

**DOI:** 10.1186/s40478-024-01755-6

**Published:** 2024-03-25

**Authors:** J. G. Nicholson, S. Cirigliano, R. Singhania, C. Haywood, M. Shahidi Dadras, M. Yoshimura, D. Vanderbilt, B. Liechty, H. A. Fine

**Affiliations:** 1 Department of Neurology, Weill Cornell Medicine, New York, NY USA; 2 Department of Pathology and Laboratory Medicine, Weill Cornell Medicine/New York-Presbyterian Hospital, New York, NY USA

## Abstract

Cerebral organoids co-cultured with patient derived glioma stem cells (GLICOs) are an experimentally tractable research tool useful for investigating the role of the human brain tumor microenvironment in glioblastoma. Here we describe long-term GLICOs, a novel model in which COs are grown from embryonic stem cell cultures containing low levels of GSCs and tumor development is monitored over extended durations (ltGLICOs). Single-cell profiling of ltGLICOs revealed an unexpectedly long latency period prior to GSC expansion, and that normal organoid development was unimpaired by the presence of low numbers of GSCs. However, as organoids age they experience chronic hypoxia and oxidative stress which remodels the tumor microenvironment to promote GSC expansion. Receptor-ligand modelling identified astrocytes, which secreted various pro-tumorigenic ligands including FGF1, as the primary cell type for GSC crosstalk and single-cell multi-omic analysis revealed these astrocytes were under the control of ischemic regulatory networks. Functional validation confirmed hypoxia as a driver of pro-tumorigenic astrocytic ligand secretion and that GSC expansion was accelerated by pharmacological induction of oxidative stress. When controlled for genotype, the close association between glioma aggressiveness and patient age has very few proposed biological explanations. Our findings indicate that age-associated increases in cerebral vascular insufficiency and associated regional chronic cerebral hypoxia may contribute to this phenomenon.

**Supplementary Information:**

The online version contains supplementary material available at 10.1186/s40478-024-01755-6.

## Introduction

The human brain is a uniquely complex organ, with vast cellular diversity, intricate cytoarchitecture and macroscopic functional regionalization, all of which emerge due to precise spatiotemporal gene expression patterns that occur during development. Remarkably, these processes can be modelled in vitro to produce human cerebral organoids (COs), which have now become invaluable tools for researching neuro-development and disease. COs are grown from pluripotent stem cells, using minimal external stimuli to allow for self-organization and spontaneous development into forebrain, midbrain and hindbrain cellular lineages [1]. Comparison to human brains reveals highly concordant developmental trajectories, with COs reaching a level of maturation equivalent to approximately 24 weeks post conception after 6 months in culture [2]. However, CO’s fidelity to developing brains is limited by their lack of non-neuroectodermal cell lineages and the absence of a functional vasculature which reduces cellular access to supplies of oxygen and nutrients. This leads to the ectopic activation of hypoxia-associated cellular stress pathways, which impairs cell subtype specification and overall CO size relative to in vivo development [3]. This has prompted several groups to develop protocols to address organoid size-imposed oxygen and nutrient diffusion limits, such as organoid slicing and air–liquid interface culture which achieved increased levels of maturation in neuronal cells [4] or in vivo transplantation of COs which led to vascularization [5]. Similarly, work on vascularized organoids of all varieties remains a field of significant interest [6, 7].

COs were first applied to the study of genetically inherited [1, 8] and infectious-disease-driven [9] neurodevelopmental disorders. More recently, however, COs have also been adapted to brain cancer research and in particular gliomas [10]. Glioblastoma (GBM) is the most common and aggressive subtype of glioma with a median survival rate of only 15 months [11]. GBM is a highly heterogenous tumor [12], which harbors a population of glioma stem cells (GSCs) that are thought to underpin the majority of tumor growth, therapeutic resistance and recurrence [13, 14]. COs present a key advantage for the study of glioma in that they can provide a human-specific brain microenvironment, which we have shown to be essential for maintenance of patient-derived human glioma stem cell (GSC) biology and cell state heterogeneity [15–17].

To date CO-GSC co-culture protocols have made use of a high starting number of GSCs (100–200 K) and assayed tumor biology over short experiments (1–2 weeks) [15]. Large numbers of colocalized GSCs, however, promote proliferation and survival, in part through autocrine signaling [18–22], making CO glioma models more representative of established and growing later stage GBMs. Here, we describe a novel model system that more closely recapitulates early gliomagenesis, or recurrence after surgery, both situations in which small numbers of GSCs must survive and grow with little autocrine signaling. By starting out with very low GSC numbers we can additionally query how GSC-microenvironment interactions change over extended periods of time, potentially shedding light on the early stages of in vivo tumorigenesis in patients.

## Results

**Fig. 1:**
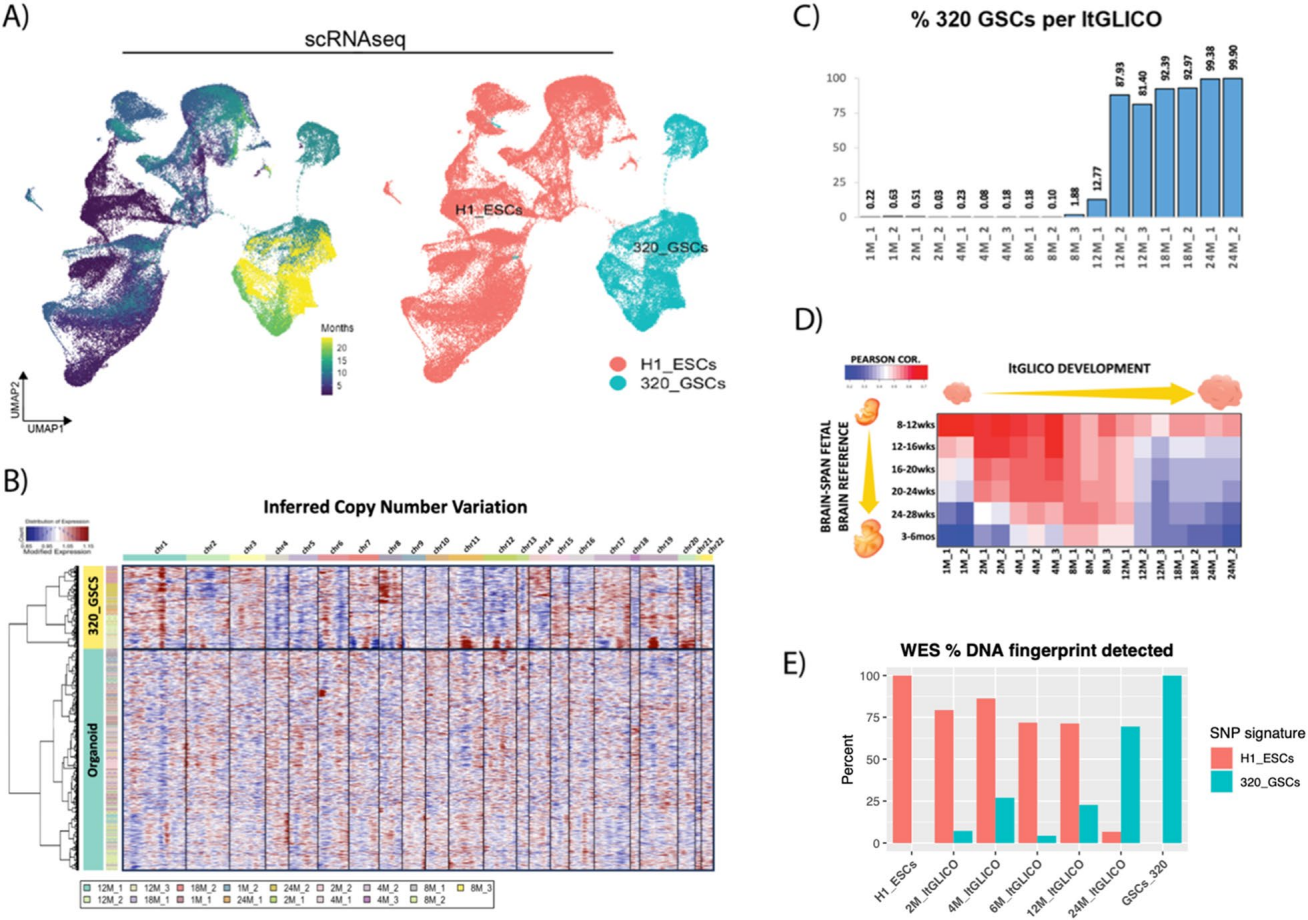
*In silcio* genotyping reveals extended latency period before GSC expansion in longterm-GLICOs. **a** UMAPs of the ageing ltGLICO cohort (spanning 1–24 months) colored by age (left), and genotype (right). **b** Inferred copy number plot separates ltGLICO cells according to genotype. **c** Proportion of 320-GSCs cells per ltGLICO sample. **d** Mean Pearson correlations of ltGLICO pseudo-bulk transcriptomes and primary human fetal brain tissue from the BrainSpan dataset grouped by age (weeks post conception). **e** Proportion of H1-ESC and 320-GSC specific SNP fingerprints detected in controls and ltGLICO samples

We, and others, have previously shown the utility of GSC—human CO co-culture models for investigating GBM biology. Here, we describe a novel model we call long-term glioma cerebral organoids (ltGLICOs)—in which new COs were generated from mixed cultures of H1-ESC containing low levels (< 1:1000) of 320-GSCs (Additional file 1: Fig. S1A). 320-GSCs were derived in-house from an IDH-wt mesenchymal subtype primary GBM patient surgical sample, and thoroughly characterized in our previous studies [15–17]. To monitor the tumorigenic process, ltGLICOs spanning 1–24 months in culture (n = 17) (Additional file 1: Fig. S1B), were profiled by scRNAseq and in silico detection of 320-GSCs was performed (see methods). This strategy was able to unambiguously genotype > 99% of all cells (Additional file 1: Fig. S1), with the remaining cells identified according to nearest-neighbor clustering (Additional file 1: Fig. S1D–E). UMAP projection showed clear separation of cells by their genotype (Fig. 1a), which was further corroborated by inferred copy number variation analysis (Fig. 1b). Intriguingly, although GSCs were detected in all ltGLICOs profiled (Fig. 1d and Additional file 1: S1F), growth did not appear to be linear with age, rather ltGLICOs had either very low levels of detectable GSCs or they had undergone near-total replacement of CO cells with 320-GSCs. We observed that 2/6 at 8–9 months, 4/6 samples at 12 months, and all samples beyond 18 months had undergone this change. To investigate the extent to which 320-GSCs disrupted normal CO development we aggregated data across all cells to test how ltGLICO pseudo-bulk transcriptomes compared to different fetal brain developmental stages recorded in the BrainSpan atlas (Fig. 1d). We saw clear evidence of progressive maturation, with ltGLICOs correlating with later developmental stages as they aged, reaching a peak of maturation between 8 and 12 months by which point they showed similarity with fetal brain from late gestational stages and early neonates. However, in those ltGLICOs that had undergone the oncogenic expansion of the 320-GSC population, this close correlation to developing brains was lost.

In addition to transcriptomic analyses, we profiled 5 ltGLICOs spanning 2–24 months as well as H1-ESCs (from which the COs were derived) and 320-GSC controls by whole exome sequencing (WES) to determine the stage at which 320-GSC mutations become detectable in ltGLICO bulk DNA extracts. Mutually exclusive germline single nucleotide polymorphism (SNP) DNA ‘fingerprints’ were generated from comparison of H1-ESC and 320-GSC genomic profiles, and ltGLICO samples were assayed for the proportion of each genotype-specific DNA fingerprint that was detected (Fig. 1e). In concordance with the single-cell findings, some 320-GSC specific SNPs were detectable in all ltGLICOs profiled, and by 24 months H1-ESC DNA had been largely replaced by 320-GSCs DNA.

To accompany our sequencing analyses, we also performed a neuropathological assessment of ltGLICO sections across the aging cohort (Additional file 1: Fig. S2A). H&E-staining showed that young ltGLICOs were macroscopically normal, with clear embryonic neural rosettes that dissipated over time, glial cells that increase with time, choroid plexus epithelium and pigmented retinal epithelium as previously shown for COs in the literature. By 9 months, however, we observed the emergence of aberrant features in the central regions of ltGLICOs. By 18–24 months, ltGLICOs were entirely replaced by cells with frankly malignant histologic features, including cells with marked nuclear pleomorphism, cells with high nuclear-to-cytoplasmic ratios, and nuclear molding. The presence of spontaneous necrosis and frequent apoptotic bodies was consistent with high-grade histology, which is typical of CNS W.H.O. grade IV GBM (Additional file 1: Fig. S2B). Immunohistochemical staining for CD44, a marker of the mesenchymal GBM-subtype strongly expressed by 320-GSCs, showed increased expression as ltGLICOs aged. We also observed changes in the patterns of Ki67 staining, with cell proliferation largely restricted to neural rosettes of younger ltGLICOs, reduced in middle aged ltGLICOs, but then greatly increased in the oldest samples (Additional file 1: Fig. S2A). Taken together, our transcriptomic, genetic, and histological analyses of aging ltGLICOs reveal that minimally seeded 320-GSCs show a prolonged latency period prior to rapid expansion and replacement of organoid cells which occurs after approximately 8–12 months in culture. Based on these analyses we focused our attention on microenvironmental changes in the CO itself that occur over time and may help provide a permissive environment for GSC cell expansion.

**Fig. 2:**
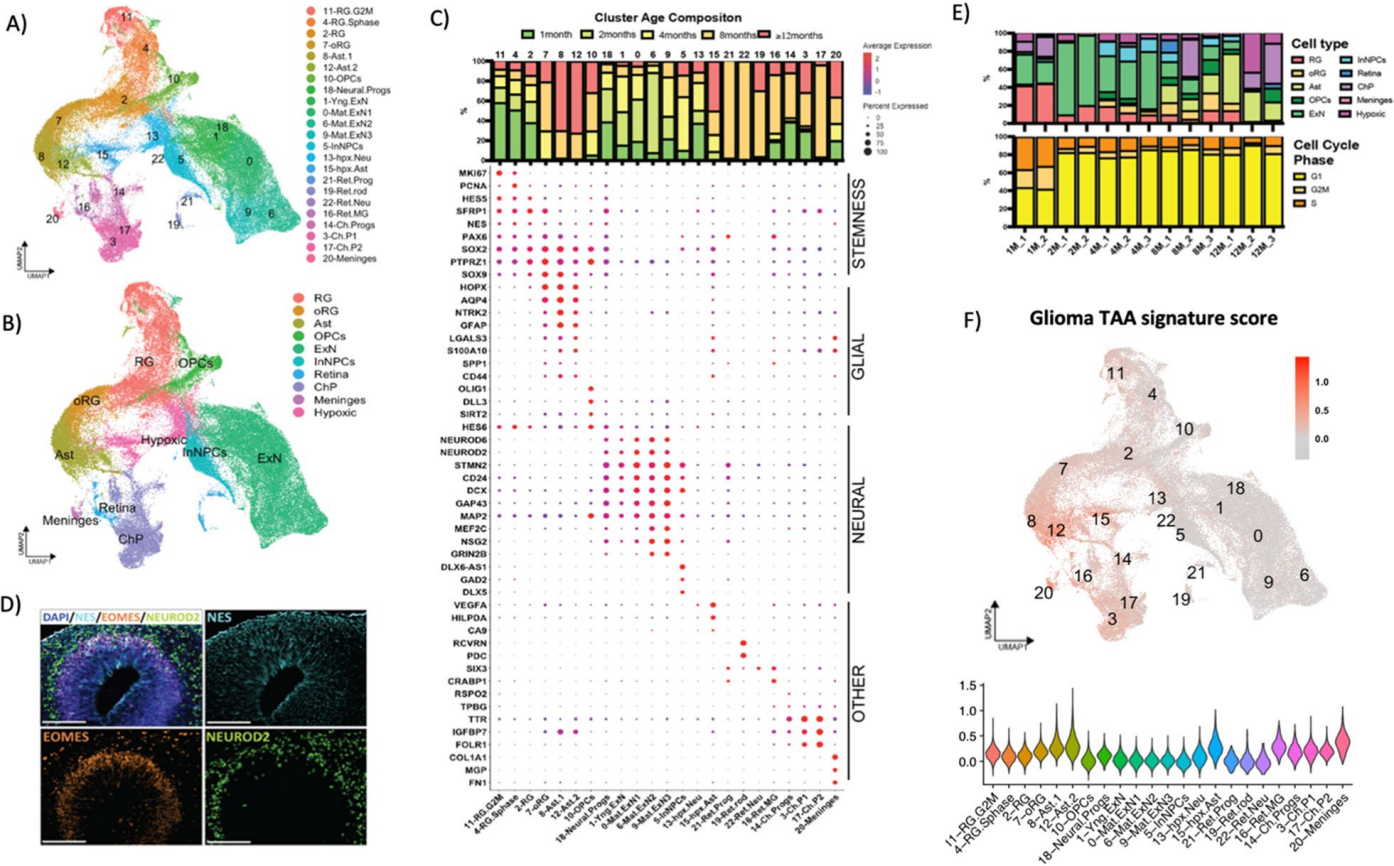
The organoid component of ltGLICOs recapitulates human neurodevelopment for up to 12 months. **a** UMAP of the CO cells from the ageing ltGLICO cohort colored by cell subtype clusters. **b** UMAP of the CO cells from the ageing ltGLICO cohort colored by cell type. **c** Dot plot of cluster marker gene expression used for cell type classification. Top; Cluster cell proportion by ltGLICO sample age. **d** Multiplex immunofluorescence staining of a representative neural rosette from a 1-month-old CO. DAPI; blue, NESTIN; cyan, EOMES; orange, NEUROD2; green. Scale bar = 100 µm. **e** Cell type and cell cycle stage proportions identified in each ltGLICO sample. **f** UMAP and Violin plot showing glioma tumor associated astrocytes (TAA) gene signature scores in single cells

To better understand how the organoid component of the ltGLICOs developed over time, H1-derived cells were subset, new UMAPs were projected, and cell clusters were identified based on known marker genes (Fig. 2a–c). 1-month-old ltGLICOs harbored numerous neural rosettes (Fig. 2d) and were highly enriched for clusters expressing markers of proliferative radial glia (C.11, 4 & 2: *MKI67*, *NES, PAX6, HES5*). 2-month-old ltGLICOs were defined by a wave of neurogenesis with expansion in the pool of newborn excitatory neurons (C.1, 18 & 0: *NEUROD2*, *DCX, CD24*), and clusters expressing transcription factors and synaptic ionotropic receptor genes associated with more mature neurons (C.9 & 6: *MEF2C, GRIN2*A). By 4 months, ltGLICOs harbor a population of midbrain inhibitory interneuron progenitor cells (InN.NPCs), that specifically express distal-less homeobox family transcription factors (C5: *DLX2, DLX5, DLX6-AS1*). The initial neurogenesis was subsequently followed by a wave of gliogenesis with the first appearance of oligodendrocytes, and their precursors (C.10: *OLIG1, DLL3, SIRT2*) in 4-month-old ltGLICOs, concurrent with expansion of outer radial glia and astrocytes (C.7, 8: *HOPX, GFAP, AQP4*). This expansion of glial cell types was even more apparent in 8-month and ≥ 12-month-old ltGLICOs (Additional file 1: Fig. S3A), by which point a new population of astrocytes had emerged. This cluster maintained expression of astrocyte markers but also showed higher expression of genes associated with reactive gliosis and environmental stress (C.10; *LGALS3, S100A10, SPP1, CD44*). Given the concurrence of the emergence of this astrocyte population and the expansion of the 320-GSCs we scored cells for a gene signature derived from glioma-associated astrocytes [23], finding the greatest signature enrichment in this cluster (Fig. 2f). Other cell types detected included choroid plexus and meninges cells (C.14, 3, 7: *TTR, IGFBP7, FOLR1* and C.20: *COL1A1, MGP* respectively) as well as three different retina cell types; retinal neural progenitors (C.21*: SIX3, SIX6, HMX1*), muller glial cells (C.16: *HMX1, CRABP1*) and rod cells (C.19: *RCVRN, PDC*). In line with previous studies [3], we also observed two clusters expressing genes associated with glycolysis (*BNIP3, PGK1*) and hypoxia (*VEGFA, HILPDA*), which corresponded to stressed neurons (C.13) and astrocytes (C.15) respectively, and increased over time as organoids grew in size. Neurodevelopment of primary fetal brain and cerebral organoids has now been thoroughly characterized and widely reported in the literature [3, 24–29]. We therefore turned to a selection of these published references to determine whether the presence of low numbers of GSCs had substantially perturbed CO development in the ltGLICO setting. Intriguingly, comparison of signatures from our assigned clusters against four primary brain and four organoid single cell datasets showed high levels of correlation between equivalent cell types (Additional file 1: Fig. S4). This suggests that the minimal levels of 320-GSC present in younger ltGLICOs has little effect on overall organoid development, which progressed in keeping with the published data.

**Fig. 3:**
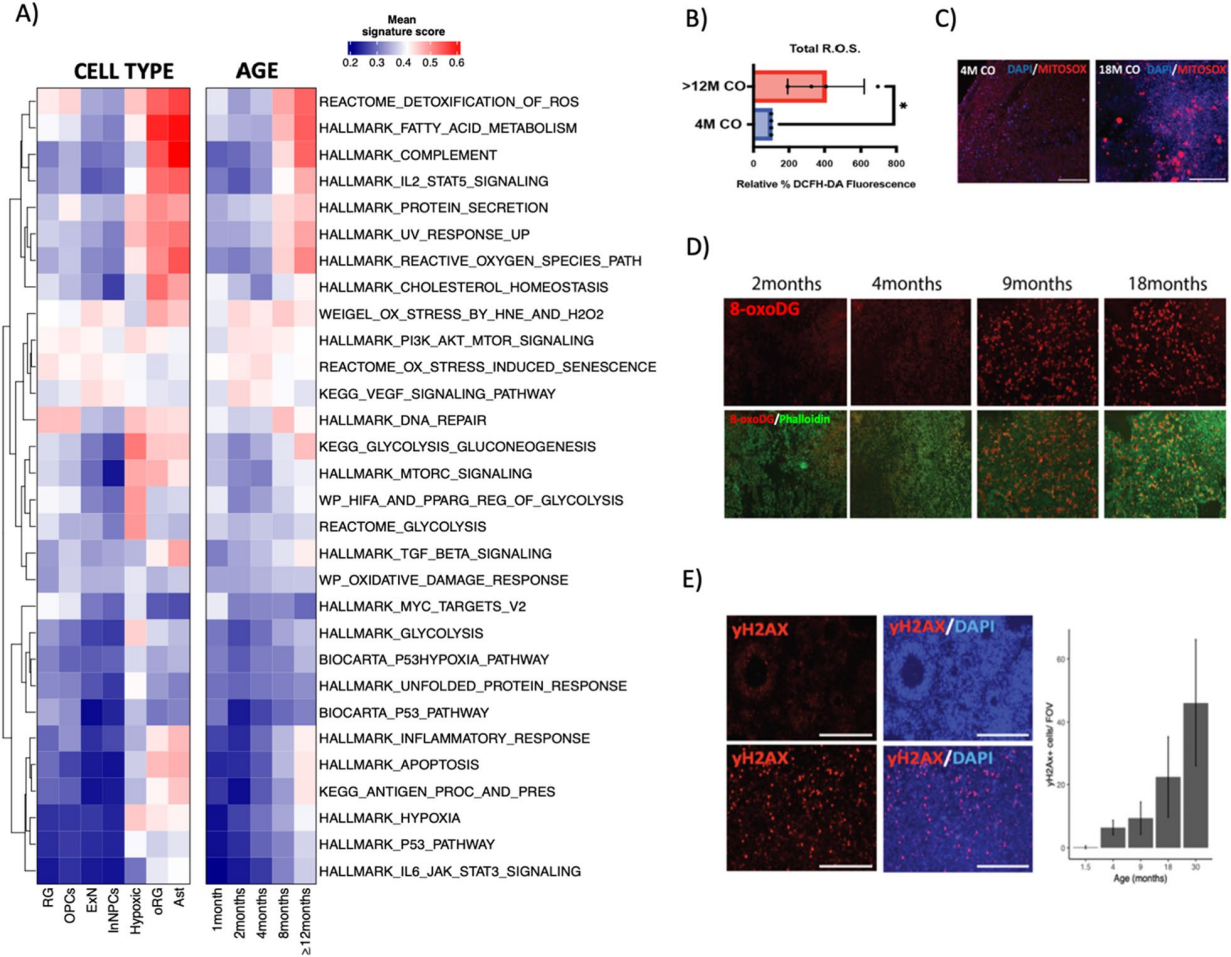
Aging ltGLICOs are characterized by increased cellular stress and oxidative DNA damage. **a** Scaled cell stress signature scores in H1-derived ltGLICO cells grouped by cell type and sample age. **b** Quantification of total ROS levels measured by 2′,7′-dichlorodihydrofluorescein diacetate (H2DCFDA)—assay. Graph shows mean + s.e.m. from four biological replicates. *p*-value = 0.0286, Mann–Whitney test. **c** Mitosox-Red staining for mitochondrial reactive oxygen species (ROS) levels in representative sections from young (4 month) and old (12 month) ltGLICOs; MitoSOX (red), and DAPI (Blue). **d** Immunofluorescence staining of ltGLICOs for 8-oxoDG (8-oxoDG in red, Phalloidin in green), representative images from a 2, 4, 9 and 18 month ltGLICOs. **e** Immunofluorescence staining of ltGLICOs for phospho-gamma-H2AX (yH2AX in red, DAPI in blue), representative images from a young (1.5 months) and old (18 month) ltGLICOs. Right, quantification of yH2AX positive cells per field of view at each CO age (plot shows mean + s.e.m.; 3 technical replicates). Scalebar = 200 µm

Cerebral organoids accurately recapitulate many aspects of human brain development [2], yet imperfect culture conditions, and the absence of functioning vasculature can lead to ectopic activation of cellular stress pathways [3]. We therefore scored ltGLICO cells for gene signatures associated with cellular stress and found that signature scores for hypoxia and glycolysis increased with ltGLICO age (Fig. 3a). As COs age they increase in size and become more densely cellular, which likely drives the steady increase in hypoxia that we confirmed by immunohistochemical staining for Carbonic Anhydrase 9 (Additional file 1: Fig. S3B). Chronic hypoxia has previously been linked to mitochondrial dysfunction and the generation of reactive oxygen species (ROS). Consistent with this phenomenon we saw a steady increase with age in signature scores associated with oxidative stress and the clearance of ROS, that was particular evident in astrocytes (Fig. 3a). This increase in ROS levels in aged ltGLICOs was validated by biochemical measurement of total ROS (Fig. 3b) and staining for mitochondrial ROS (Fig. 3c). When DNA is exposed to ROS, guanine can be modified to 8-oxo-dG, ultimately giving rise to DNA double strand breaks [30]. To detect whether elevated ROS levels were causing damage to ltGLICO cells we performed immunofluorescence against 8-oxo-dG and phosphorylated-yH2X (Fig. 3d–e). This showed marked increases in 8-oxo-dG and phosphorylated-yH2X over time indicating that oxidative stress, and its associated DNA damage, increases as ltGLICOs age. Whilst chronic hypoxia represents the most likely driver of oxidative stress, it is also possible that the presence of 320-GSCs in the microenvironment of CO cells may in some way contribute to this process. To confirm that CO hypoxia and oxidative stress increases with age in the absence of 320-GSCs we turned to published RNAseq data from 62 organoids generated from 6 different cell lines (Additional file 1: Fig. S3C). Transcriptomic signature scoring indicated that progressive oxidative stress was common to other brain organoid culture protocols in the absence of 320-GSCs.

**Fig. 4:**
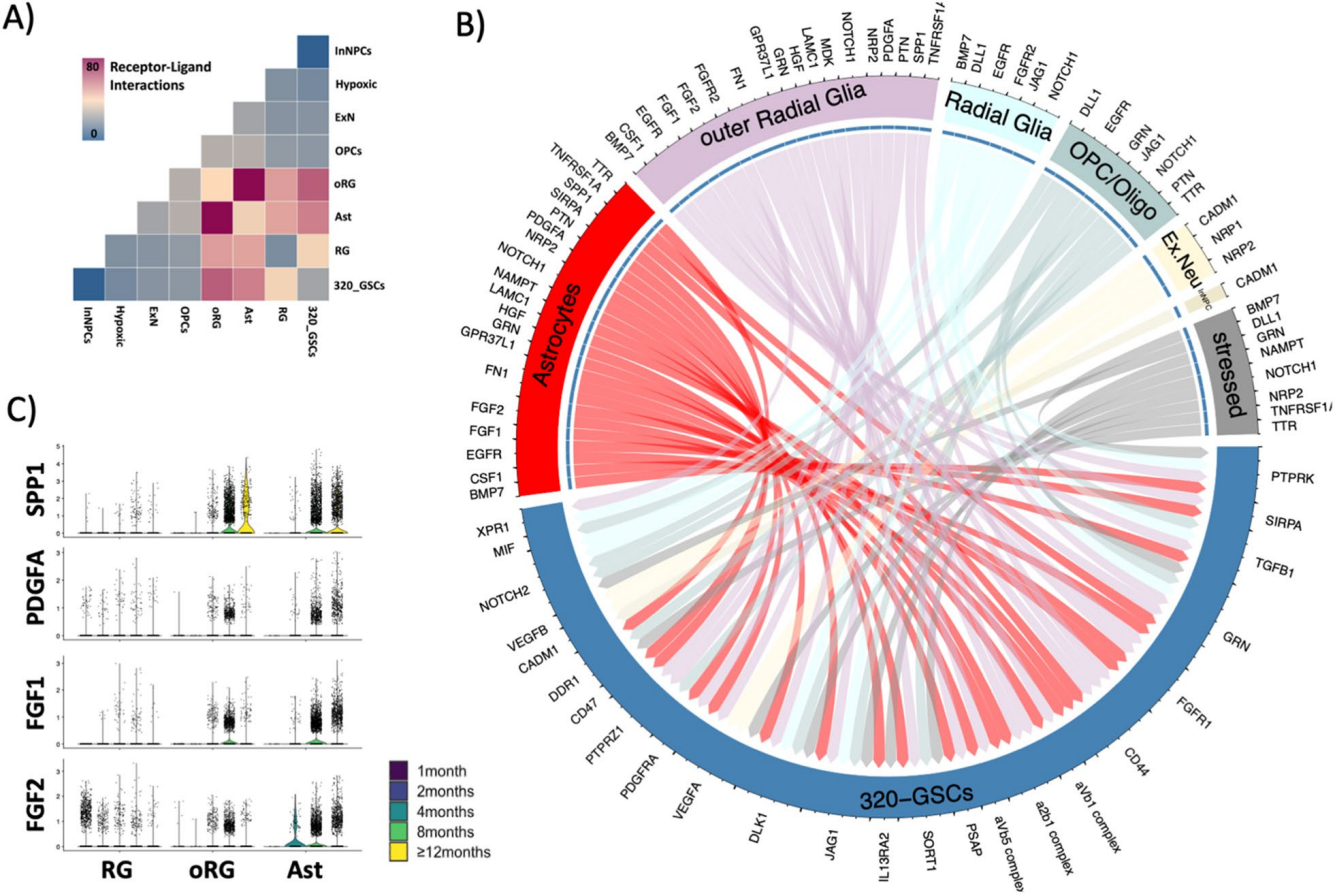
Receptor ligand modelling uncovers astrocyte expression of candidate pro-tumorigenic ligands. **a** Heatmap showing the total number of interactions between cell types in ltGLICO dataset obtained with CellPhoneDB. **b** Circos plot showing interaction between ligands secreted by organoid cell types and receptors expressed by 320-GSCs. **c** Violin plot showing expression of oxidative stress associated ligands (SPP1, PDGFA, FGF1, FGF2) in glial cells

We next performed receptor-ligand modelling to query our scRNAseq data for candidate microenvironmental factors that may promote GSC growth (Fig. 4). Notably, 320-GSCs demonstrated their highest number of putative receptor-ligand interactions with organoid cells of the astroglial lineage including outer radial glia and astrocytes cells (Fig. 4a). Among the organoid-secreted ligands were numerous microenvironmental factors previously reported to play a pro-tumorigenic role in glioma (Fig. 4b). These included SPP1, FGF1/2, PDGFA and PTN [31–35] (Fig. 4c), several of these ligands are known to be upregulated under conditions of hypoxia and oxidative stress [36–39]. Furthermore, their expression levels peak during months 8–12 of ltGLICO culture coincident with the growth of the GSCs. Based on these findings we hypothesized that progressive hypoxia and oxidative stress may remodel the ltGLICO microenvironment to promote GSC growth by increasing the secretion of pro-tumorigenic ligands by glial cells.

**Fig. 5:**
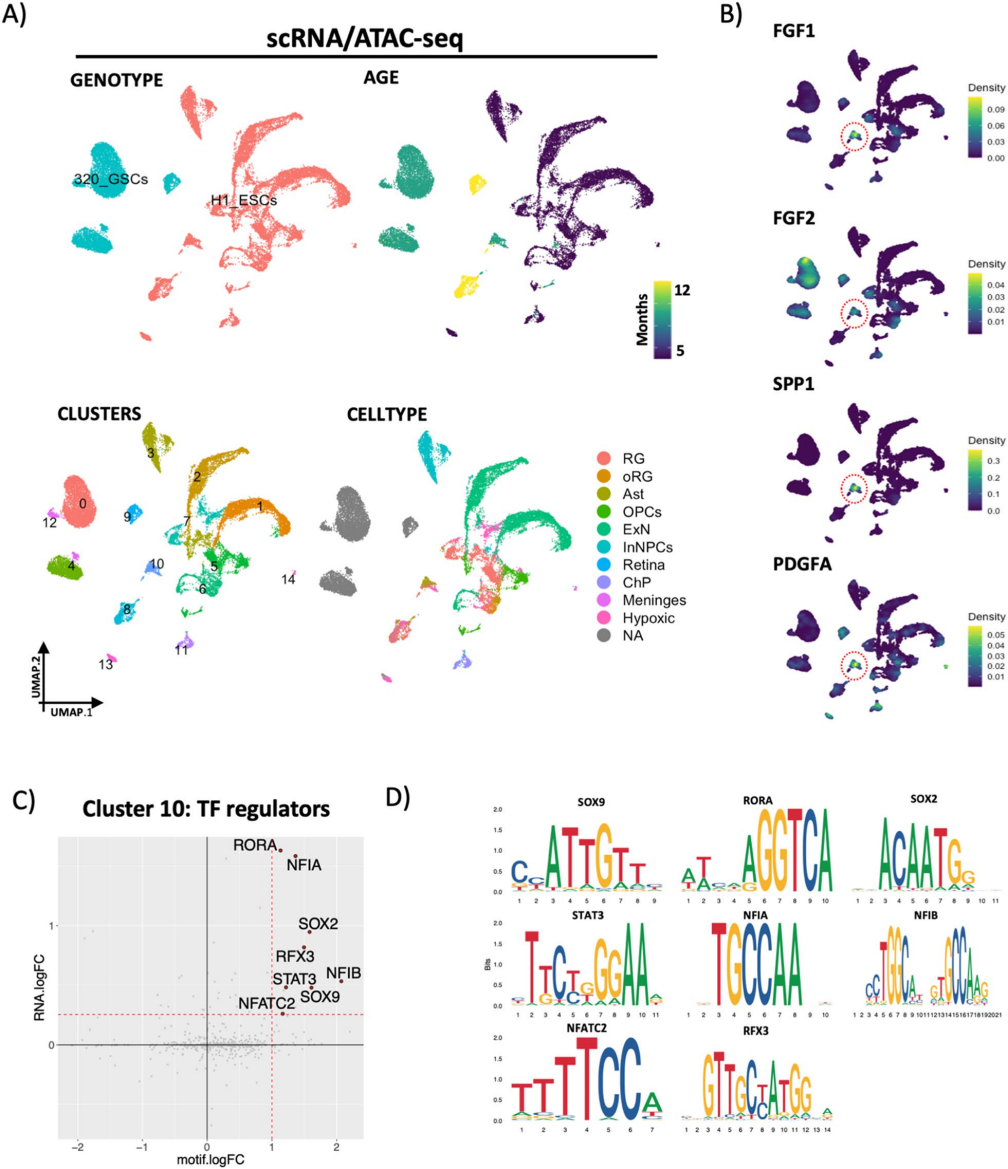
ltGLICO astrocytes are defined by regulators characteristic of reactive gliosis and ischemia. **a** UMAPs calculated on combined (weighted nearest neighbors) RNA and ATAC single cell data from 5 to 12 month ltGLICOs colored by genotype, age, clusters and cell type. **b** Expression density plots of pro-tumorigenic ligands (FGF1, FGF2, PDEFA and SPP1) in ltGLICOs. **c** Differential expression of transcription factors and enrichment of their associated motifs in cluster 10 astrocytes. **d** Motif plots of enriched regulators

To better understand the regulatory mechanisms underpinning a putative pro-tumorigenic role for hypoxic astrocytes, we next performed single-cell multiome-seq (scATAC and scRNA in the same cells) on a cohort of 8 ltGLICOs spanning 5–12 months. Computationally identified 320-GSCs were well separated from organoid cells in UMAP dimensional reductions calculated on RNA and ATAC independently (Additional file 1: Fig. S5A), or together and samples showed similar long latency periods before 320-GSC expansion (Fig. 5a). We next clustered cells and transferred cell type labels from the larger scRNAseq cohort. Cluster 10 was of particular interest since it was composed of mostly astrocytes, with some hypoxic and outer radial glia cells, and was enriched for cells from 8 to 12 month GLICOs—coinciding with the expansion of 320-GSCs. Furthermore, expression analysis of candidate pro-tumorigenic ligands (FGF1, FGF2, SPP1 and PDGFA) revealed enrichment in the cluster (Fig. 5b). We next used the chromosome accessibility data to identify cluster specific enrichment for transcription factor motifs (Additional file 1: Fig. S5B)—which identified enrichment for cell-type defining transcriptional regulators in the expected clusters: 320-GSCs were enriched for AP-1 transcription factors FOSL1/2 and JUN; excitatory neurons for NEUROD1/2; Inhibitory neuron progenitors for DLX2/5 and OPCs for ASCL1. For cluster 10, we considered transcription factors that had both motif enrichment and upregulated expression for the corresponding transcription factor, reasoning that these would be the most important factors in defining this pro-tumorigenic astrocytic state (Fig. 5c, d). Among the most enriched regulators were SOX9 and NFIA, both key regulators involved in the development and maintenance of astrocytes [40, 41], and RORA which is predominately expressed in adult astrocytes [42]. Other identified regulators are involved in reactive gliosis, such as SOX2 which plays a role in astrocyte activation after traumatic brain injury [43], and STAT3 which has been widely implicated in astrocyte activation and inflammation during neurological disease [44], and under hypoxic/ischemic conditions [45–47]. Another interesting regulator identified was NFATC2, an effector of the Calcineurin/NFAT pathway which is associated with chronic hypoxia in astrocytes during cerebellar vascular disease and is upregulated in astrocytes following CNS injury and disease [48]. Together these analyses suggest that astrocytes present in ltGLICOs are maintained by a regulatory network associated with reactive gliosis that characteristic of CNS disease and under conditions of ischemia.

**Fig. 6:**
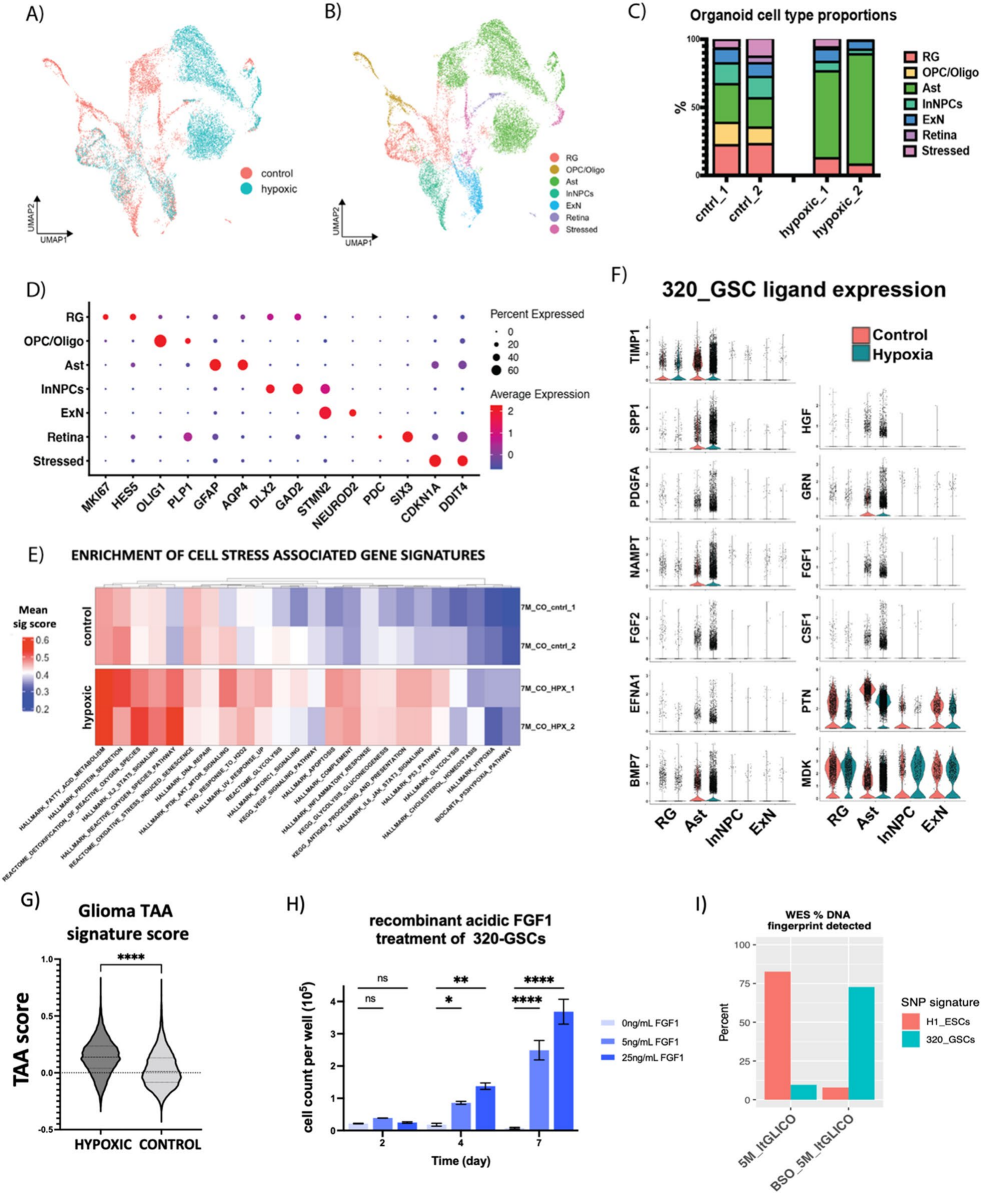
Hypoxia remodels the cerebral organoid microenvironment and increases the secretion of pro-tumorigenic ligands. **a** UMAP of hypoxic culture CO cohort colored by oxygen condition. **b** UMAP of hypoxic culture CO cohort colored by cell type. **c** Cell type proportions identified in each CO sample. **d** Dot plot of cluster marker gene expression used for cell type classification. **e** Scaled cell stress signature scores, single cells grouped sample. **f** Violin plots of candidate pro-tumorigenic ligand expression in hypoxic culture CO cohort. **g** Violin plot of glioma TAA signature scores in hypoxic vs control astrocytes. **h** Effect of recombinant acidic FGF1 on 320_GSC growth. **i** Proportion of H1-ESC and 320-GSC specific SNP fingerprints detected in control 5-month-old ltGLICO and 5-month-old ltGLICO treated for 6 weeks with 50uM Buthionine sulfoximine (BSO)

To test the theory that chronic hypoxia promoted astrocytic secretion of pro-tumorigenic ligands, and to discount the possibility that the GSCs themselves were prompting the ligand secretion, we cultured standard COs—absent any GSC component—under hypoxic conditions (5% O_2,_ n = 2) for 6 weeks and compared them to normoxic (~ 20% O_2_, n = 2) controls by scRNAseq. UMAP projections of cells colored by their oxygen culture conditions showed clear differences (Fig. 6a) and identifying cell types based on their marker gene expression (Fig. 6b–d) revealed changes in their cell type proportions. OPC/Oligos were completely absent, and InNPCs were depleted under hypoxic culture conditions likely indicating greater sensitivity to hypoxia for these cell types. By contrast, astrocytes—the primary cell type contributing to organoid-GSC crosstalk—were greatly expanded (Fig. 6c). As expected, scoring for gene signatures associated with cellular stress processes showed enrichment in hypoxic organoids (Fig. 6e). Of note was upregulation of the hallmark_protein_secretion gene set, as well as several associated with oxidative stress. This indicates that chronic hypoxia is the likely root cause of the oxidative stress phenotype seen in aging ltGLICOs, and COs more generally. Given the increase in the signature for protein secretion we next queried the major cell types present in both culture conditions (radial glial, astrocytes, excitatory neurons and Interneuron NPCs) for expression of candidate pro-tumorigenic secreted ligands (Fig. 6f). Interestingly the majority of these ligands showed upregulation in hypoxic astrocytes. Furthermore, scoring astrocytes for the glioma TAA gene signature [23] revealed that astrocytes kept under hypoxic conditions were significantly more similar to those observed in the glioma tumor microenvironment than their normoxic counterparts (Fig. 6g). Given that a pro-tumorigenic role for acidic-FGF1 has not previously been established for glioma, we evaluated the effect of recombinant FGF1 on 320-GSCs in growth factor depleted media, revealing a dose-responsive increase in 320-GSC growth (Fig. 6h). We observed similar results with a second patient derived cell line 1206-GSCs (Additional file 1: Fig. S5C).

Together these results show that exposure of COs to chronic hypoxia increases oxidative stress and the population of astrocytes that secrete pro-tumorigenic ligands. Based on these findings we next tested whether pharmacological modulation of microenvironmental oxidative stress in ltGLICOs could accelerate GSC growth. ltGLICOs were cultured in 50uM buthionine sulphoximine (BSO), an inhibitor of glutathione-synthetase an enzyme critical for the clearance of ROS which we found to be upregulated in older ltGLICO astrocytes (Additional file 1: Fig. S5D). After 6 weeks treatment, we performed WES on a BSO-treated 5 month ltGLICO and a control sample. Comparison of the detected proportion of H1-320 and GSC-320 specific DNA fingerprints revealed a major increase in the proportion of 320-GSC DNA detected in the BSO treated ltGLICO, with Glioma driver mutations clearly detectable, whereas they remained below the threshold of detection in the control sample (Fig. 6i).

## Discussion

Here we present ltGLICO, an adapted GSC-human CO co-culture model in which COs are generated containing minimal levels of tumor initiating GSCs and then monitored for extended durations. This contrasts with our typical GLICO model in which several orders of magnitude more GSCs are used for tumor initiation and the resultant tumors are assayed over shorter time periods. GSC autocrine signaling, and modulation of their microenvironment are well known to promote glioma growth [18–21]. We therefore propose the sparse seeding of ltGLICOs to more closely model early glioma tumorigenesis or the residual infiltrating tumor front following gross total resection of a recurrent glioma where GSCs do not yet reside in an environment optimized for tumor growth. Intriguingly, despite the early seeding of ltGLICOs with GSCs we observed a substantial latency period of up to 12 months before to GSC expansion and replacement of H1-ESC derived organoid cells in ltGLICOs occurred. Importantly, the time course of GSC expansion remained consistent and reproducible across numerous starting batches of ltGLICOs. This extended delay is reminiscent of a study of human glioma that predicted the emergence of founder cells approximately 2–7 years before diagnosis, and that most tumor cell divisions result in cell death, such that only a minor fraction of cell divisions supports tumor growth [49].

The initiating GSCs in our ltGLICO model are derived from a mature glioblastoma patient, reducing the likelihood that the delay in growth is governed by time necessary for tumor clonal evolution, and indeed we did not see evidence of the acquisition of novel driver mutations in even the oldest WES samples. Instead, our findings strongly support a role for age-associated microenvironmental changes in ltGLICOs as the trigger for increased GSC growth. As COs age beyond 8 months they harbor an increasing proportion of astrocytes which according to receptor-ligand modelling have the greatest degree of crosstalk with GSCs among neuroglial cell types. Furthermore, as COs grow, they become larger and more hypoxic, which increases oxidative stress and the expression of numerous pro-tumorigenic ligands. This conclusion is supported by our experimental data demonstrating that pharmacological enhancement of oxidative stress with BSO accelerates GSC expansion in ltGLICOs. In addition to several well-known pro-tumorigenic ligands secreted in ltGLICOs by hypoxic astrocytes, we also uncovered FGF1, and then verified its positive effect on GSC growth and survival in vitro using recombinant protein and two independent patient derived GSC cell lines. FGF signaling is complex and context dependent, and a limitation of our in vitro growth assays was that they were not performed under hypoxic culture conditions to mimic the environment of an aged ltGLICO which may have influenced the effect of FGF1. However, it has previously been reported that chronic hypoxia increases expression of the FGFR1 receptor [50], and indeed we observed increases in 320-GSCs FGFR1 expression over time as ltGLICOs become more hypoxic (Additional file 1: Fig. S5D), so its possible that by conducting the FGF1 growth assays under normoxic conditions we do not capture the full pro-tumorigenic effect of the FGF1 in ltGLICOs.

Cerebral organoids are useful but imperfect models for glioblastoma research, as they lack immune cells or a functional vasculature. This absence of vasculature is of key importance for the translational relevance of our findings, since in our system the root cause of pro-tumorigenic cerebral hypoxia is an experimental artefact. However, as the human brain ages the incidence of cerebral vascular insufficiency and disease increases, leading to areas of relative chronic cerebral hypoxia. Additionally, over time, cells accrue damaged mitochondria which are less efficient and generate higher levels of ROS that result in oxidative stress and further mitochondrial damage, a vicious cycle associated with several neurodegenerative diseases [51]. To date there is little understanding of why IDH-wt GBM poor prognosis, on a genotype-for-genotype matched basis, is associated with advanced age, but our data point towards hypoxia and oxidative stress as two possible contributing factors that help explain this phenomenon. Consistent with this theory, polymorphisms in glutathione S-transferase and superoxide dismutase 1, two enzymes involved in ROS clearance, are among the few associated with increased glioma incidence [52–54]. Thus, it is interesting to speculate that decreasing cerebral perfusion and resultant relative localized cerebral hypoxia or general mitochondrial decline may help generate a microenvironment that promotes GSC growth in aging patients. Finally, it is known that a long-term effect of radiation therapy—the most standard treatment for malignant gliomas—is the destruction of normal cerebral microvessels thereby inducing areas of relative ischemia and hypoxia. If true, might radiation therapy be “conditioning” the local cerebral microenvironment in a way conducive to eventual aggressive tumor recurrence?

## Materials and methods

### Experimental model and subject details

#### Patient-derived GSCs

320- and 1206-GSC cell lines were derived as previously described [15–17] and reused here. Briefly, after informed consent was obtained, tumor samples classified as glioblastoma, based on the World Health Organization (WHO) criteria, were collected from patients undergoing surgical treatment at the National Institutes of Health (NIH) or from Weill Cornell Medicine/New York Presbyterian Hospital in accordance with the appropriate Institutional Review Boards. Within 1–3 h after surgical removal, tumors were washed in PBS and enzymatically dissociated into single cells. Tumor cells were cultured in NBE medium consisting of neurobasal medium (Thermo Fisher Scientific), N2 and B27 supplements (Thermo Fisher Scientific), and human recombinant bFGF and EGF (25 ng/mL each; R&D Systems) plus Heparin sodium and L-Glutamine. Regular mycoplasma screening was performed using the MycoAlert Detection Kit (Lonza Inc.).

#### Human ESCs

NIH-registered human H1 (WA01) embryonic stem cells were purchased from WiCell Research Institute, Inc. and maintained in mTeSR1 medium (STEMCELL Technologies).

### Method details

#### Cerebral organoid and long-term-GLICO culture

Microfilament-engineered Cerebral Organoids and ltGLICOs were prepared as previously described [55] and maintained on an orbital shaker with bi-weekly media changes. The initial mixing of H1-ESCs and 320-GSCs and subsequent generation of ltGLICOs was inadvertent. However, given the experimental advantages of a low-seeded GSC-Co co-culture model, we took the opportunity to continue with their biological characterization. The initial seeding ratio of 320-GSCs was determined to be < 1:1000 based on a PCR using primers targeting an RFP construct present in the 320-GSCs (Additional file 1: Fig. S1A).

#### Immunofluorescence of COs

ltGLICOs were fixed in 4% paraformaldehyde for 45 min at room temperature followed by three PBS washes for 10 min each and then embedded in paraffin. Sections of 4 um were obtained using microtome on poly-lysine–coated slides. Following deparaffinization and rehydration, antigen retrieval was performed by submerging the slides in Trilogy solution (Sigma, 920P) and heating in a pressure cooker for 15 min. Sections were permeabilized for 20 min with PBS/0.5% Triton X-100 at room temperature and blocked 1 h with PBS/3% BSA. Each section was incubated overnight at 4 °C with primary antibodies against phosphorylated-yH2AX (Cell Signaling #2577; 1:400), followed by an incubation with the secondary antibody coupled Alexa 568 (Invitrogen #A11036; 1:1000) for 1 h at room temperature. Nuclei were counterstained with DAPI. For 8-oxo-DG immunofluorescence, deparaffinized and rehydrated Sections were incubated for 15 min at 37 °C in 50 µL of Proteinase K (10 µg/ml), washed in 1X PBS for 5 min, and incubated in 200 µL of a buffer solution (100 µg/ml RNase A, 150 mM NaCl, and 15 mM sodium citrate) for 1 h at 37 °C. Slides were washed in 1X PBS twice for 10 min and blocked in 10% Normal Goat Serum for 1 h at room temperature. Slides were then incubated overnight at 4 °C with a 1:250 dilution of 8-oxo-DG primary antibody (4354-MC-050; Novus Biologicals, Centennial, Colorado) in 0.1% BSA in 1X PBS. The following day, sections were incubated with secondary antibody Alexa 568 (Invitrogen #A11036; 1:1000) for 1 h at room temperature. Actin filaments were counterstained with Phallodidin-iFluor 488 (ab176753; Abcam, Cambridge, UK). Images were obtained using an epifluorescence microscope, processed, and analyzed using the Fiji software.

For multiplex immunofluorescence, sections from ltGLICO aged 1 month processed as above, were analyzed using the Vectra Polaris® Automated Quantitative Pathology Imaging System, with the following antibodies: Rabbit polyclonal anti-GFAP (Z033429-2, Dako); Mouse anti-Nestin (MAB5326, Millipore); Rabbit polyclonal anti-EOMES (ab23345, Abcam); Rabbit anti-NEUROD2 (MA536147, ThermoFisher).

### Reactive oxygen species quantification

COs were incubated with 5 µM MitoSOX-Red superoxide indicator (ThermoFisher, cat number: M36008) in culture media for 10 min at 37 °C, and then fixed in 4% PFA for 1 h at 4 °C. Nuclei were stained with DAPI (Invitrogen) during the last 10 min of PFA fixation. After PFA fixation COs were washed with Hanks' Balanced Salt Solution (HBSS) without phenol red. MitoSOX-Red staining was analyzed with Zeiss LSM 880 Laser Scanning Confocal Microscope. For total intracellular ROS quantification, COs were incubated with 2.5 µM DCFH-DA (ThermoFisher Scientific, cat number: D399) in HBSS without phenol red for 30 min at 37 °C. Intracellular esterases convert DCFH-DA to 2′,7′-dichlorodihydrofluorescein that in turn is converted into 2′,7′-dichlorofluorescein when oxidized by H2O2. COs were lysed in RIPA buffer kept at 4 °C followed by fluorimetry. Fluorescence was measured in a Microplate Fluorescence Reader Promega GloMax (excitation wavelength, 485 nm; emission wavelength, 520 nm). Cell auto-fluorescence (produced by cells incubated with HBSS without fluorometric probe) was subtracted. Protein concentration of cell lysates was determined using Pierce BCA protein kit (ThermoFisher, cat number: 23227). Results were calculated at fluorescence units per μg protein and then expressed as percentage of control (young organoids).

### Recombinant acidic FGF1 growth assay

For GSC proliferation assays, 320-GSCs and 1206-GSCs were plated in a 6-well plate at a density of 200,000 cells per well in 2 mL growth factor depleted NBE media (without EGF or basic FGF). After 2 h of incubation, cells were treated with either 0, 5, or 25 ng/mL Human FGF-acidic/FGF1 Recombinant Protein (27,398; *Cell Signalling Technology*). Cells were then harvested and counted using Trypan Blue solution (Sigma-Aldrich) after 2, 4, and 7 days for 320 GSCs and 3, 5, and 7 days and 1206 GSCs to assess proliferation. Experiments were repeated twice with two technical replicates.

#### NGS sample preparation

For single cell RNA- and Multiome- sequencing, samples (COs & ltGLICOs) were first enzymatically dissociated into single cells (Worthington Biochemical, LK003150) then passed through a 40 um strainer and resuspended on ice-cold PBS/2%BSA, using the Trypan blue Exclusion test to confirm high cell viability. For whole exome sequencing genomic DNA was extracted from single cell suspensions using DNeasy (QIAGEN, cat number: 69504) according to the manufacturer’s instructions.

### Quantification and statistical analysis

#### scRNA-sequencing processing

scRNA-seq libraries were prepared with the Chromium Single Cell 3′ Library & Gel Bead Kit v.2 (10 × Genomics, PN-120237), and sequenced on a NextSeq 500 instrument (Illumina). The Cell Ranger 2.0.1 pipeline was used to align reads to the GRCh38 human reference genome and produce count matrices for downstream preprocessing and analysis using the Seurat v4.0 R package [56]. For quality control, cells with fewer than 750 genes detected or greater than 10% mitochondrial gene expression were removed. Ribosomal genes, and those genes detected in fewer than five cells were excluded from analyses. Expression values were library size corrected to 10,000 reads per cell and log1p transformed, with Principal component analysis (PCA) performed on the scaled data for the top 2000 variable genes. Batch correction was performed on principal components using Harmony [57]. Uniform Manifold Approximation Projection embeddings, Nearest Neighbors and cell clusters were then calculated in harmony-corrected PCA space using the default settings of Seurat’s RunUMAP(), FindNeighbors(), and FindClusters() functions. For scRNAseq analysis of hypoxic and normoxic COs no batch correction was required. Cell cluster markers were identified by Wilcoxon Rank Sum test—with Gene Ontology enrichment of cluster marker genes performed using the topGO R package. For reference signature scoring, average gene module expression was calculated for each single cell, subtracted by the aggregated expression of a random control set of features selected from the same average expression bins as the query genes [58]. G/S and G2M gene modules are included in the Seurat v4.0 package; P53, hypoxia, glycolysis and oxidative stress signatures were taken from MSigDb [59]. For receptor-ligand analysis, non-neuroglial cells (choroid plexus, retina and meninges) were removed, and cell type meta data and count data for the top 5000 most highly variable genes from remaining cells was input into CellphoneDB v2.0 [60], and run using the statistical_analysis mode. For circos plot visualization the Circlize v0.4.15 R package was used, and only those significant receptor-ligand interactions that involved tumor-microenvironment ligands and 320-GSC receptors were included in the plot.

#### scMultiome-sequencing processing

Raw multiome sequencing data were converted to fastq format and subsequently aligned to the GRCh38 genome using “cellranger-arc mkfastq” and “cellranger-arc count” (10X Genomics, v2.0.0). Paired scRNAseq and scATACseq mulit-ome data was then analyzed using the Seurat v4.0 [56] and Signac v1.10 R packages [61]. Briefly, a Seurat Object was created from the matrix.h5 and fragments.tsv.gz files, annotated with EnsDb.Hsapiens.v86, and ATAC peaks corrected by calling with macs2 (v2.2.7.1). To ensure only high quality cells were analyzed the following quality thresholds were implemented: nCount_RNA > 1000, nCount_ATAC > 1000, nucleosome_signal < 2 and TSS.enrichment > 1. Expression values were library size corrected to 10,000 reads per cell and log1p transformed, with Principal component analysis (PCA) performed on the scaled data for the top 2000 variable genes. ATAC counts were processed using latent semantic indexing (LSI). Joint scRNA and scATAC UMAP visualization and clustering were then performed using the weighted nearest neighbor methods in Seurat v4. For annotating single cells we used the processed scRNAseq ltGLICO R object (Fig. 2) as a reference an the default settings of Seurat v4’s FindTransferAnchors(), TransferData() and AddMetaData() functions. Expression density plots of pro-tumorigenic ligands were generated using Nebulosa v1.10 [62]. For identification of cell type specific regulators, we first scanned ATAC peaks for DNA motifs present in in the JASPAR2020 database, single-cell motif activity z-scores were calculated using chromVAR [63]. Finally we used presto [64] to perform Wilcox rank sum tests and calculated differential gene expression and chromVAR motif accessibilities between clusters.

### In silico detection of 320-GSCs

For both scRNAseq and scMultiome ltGLICO samples, in silico detection of 320-GSCs was performed using Souporcell [65], a tool designed to demultiplex mixtures of genotypes from droplet-cased scRNA-seq protocols. First we generated several merged bamfiles from cellranger gene expression outputs; for scRNAseq we grouped samples across the timecourse making one bamfile for each set of technical replicates, for scMultiome we generated a single bamfile for all samples. Next we performed reference-free demultiplexing of the merged bamfiles manually setting the output clusters to k = 2. The genotype of assigned clusters was determined by CNV analysis, and any cells for which souporcell did not assign a cluster (< 1%) were assigned to a genotype based on nearest neighbor clusters (see Additional file 1: Fig. S1D).

### Comparison to reference datasets

Pseudo-bulk transcriptomes were generated from aggregated ltGLICOs scRNAseq samples and compared to in vivo fetal and neonatal brain samples obtained from the BrainSpan cohort. The top 2000 variable genes in the reference cohort were calculated by VST estimation and used for Pearson correlation between ltGLICOs and in vivo samples. Results were grouped by reference sample age and reported as means.

scRNAseq cluster gene expression was compared against cell type signatures from four studies of developing human [3, 24, 25], and mouse brain [26], and four different neuroectodermal organoids datasets [3, 27–29]. In each case we obtained both the raw count matrices and author-defined cluster meta data, and used them to perform a Wilcoxon Rank Sum test to obtain the list of cluster marker genes for their cell types. Then, for each dataset and marker gene, cluster specificity scores were computed (mean normalized counts per cluster/total mean normalized counts)—with gene signature specificity scores compared across studies by Pearson correlation. For each pair of datasets, we restricted analyses to the intersection of their full marker gene lists.

To confirm oxidative stress increases over time in wild-type organoids we accessed 62 bulk-RNAseq from a study of cortical organoids ranging from 25 to 692 days in culture [66]. For gene set scoring in RNAseq samples, raw counts were first library size normalized to cpm and log1p transformed. Module scores for each sample were then calculated as the relative average expression for all genes within the set, minus the average expression of a random control set of features that were selected from the same expression bins as the query genes (approach described in [58]).

### Whole exome sequencing

The SureSelect Human All Exon V6 kit (Agilent) was used for exome capture on CO and spGSC samples, as well as H1 ESC and 320-GSC controls. Samples were sequenced with an Illumina NovaSeq 6000 machine, reads were mapped to the human genome build GRCh38 with Burrows-Wheeler Aligner [67], and bamfiles were pre-processed using the Genome Analysis Toolkit (GATK [68]). We then employed GATK best practices pipelines for somatic short variant (SNVs + Indels) and copy number variant discovery. Somatic SNVs were inferred with MuTect2 [69] and germline SNPs were inferred using HaplotypeCaller [70] with annotation performed using Funcotator.

### Supplementary Information

**Additional file 1**. **Figure S1**: Identification of 320-GSCs in ltGLICOs. **Figure S2**: Histopathological analysis of the ltGLICO cohort. **Figure S3**: Aging organoids show increased hypoxia and oxidative stress, **Figure S4**: Correlation of ltGLICO organoid cells to brain organoid and primary fetal brain reference datasets. **Figure S5**: scMulti-ome analysis of 5-12month ltGLICOs.

## Data Availability

Data generated in the study have been deposited in public repositories and are available under the following accession numbers: scRNA/Multiome-seq GSE210736, whole exome sequencing PRJNA866851.

## Code availability

Custom code used in this study will be made available from the corresponding authors upon reasonable request.
