# Supplementary material for: Chronic hypoxia remodels the tumor microenvironment to support glioma stem cell growth
Source: Acta Neuropathol Commun. 2024 Mar 25;12:46. doi: 10.1186/s40478-024-01755-6 (PMC10964514; doi:10.1186/s40478-024-01755-6)
Supplement: Supplementary file 1 — Additional file 1. Figure S1: Identification of 320-GSCs in ltGLICOs. Figure S2: Histopathological analysis of the ltGLICO cohort. Figure S3: Aging organoids show increased hypoxia and oxidative stress, Figure S4: Correlation of ltGLICO organoid cells to brain organoid and primary fetal brain reference datasets. Figure S5: scMulti-ome analysis of 5-12month ltGLICOs. [file 40478_2024_1755_MOESM1_ESM.docx]

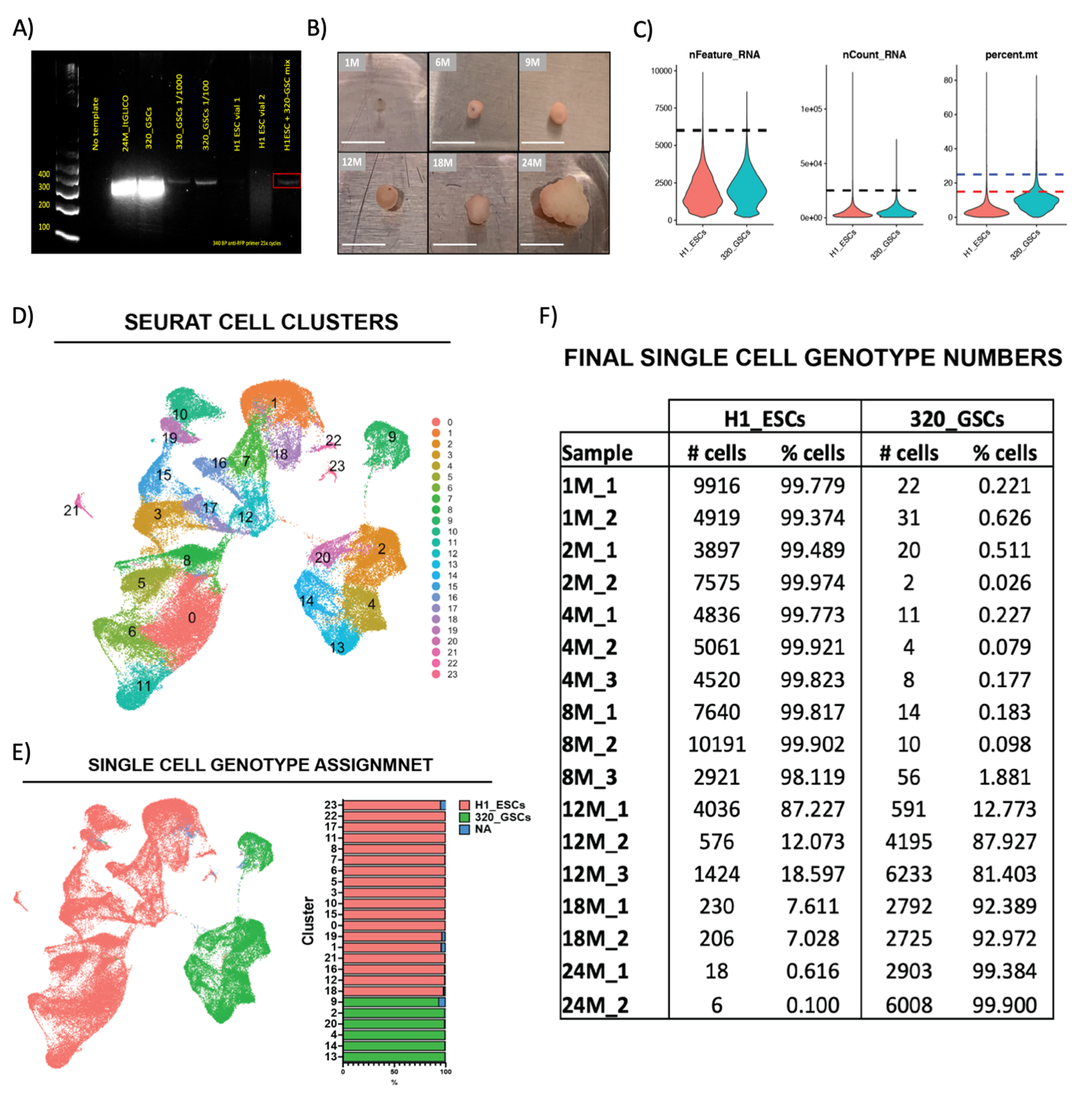


**Figure S1: Identification of 320-GSCs in ltGLICOs**

1. Anti-RFP PCR used for the detection of 320-GSCs in H1-ESC ltGLICO seeding mixture. Genomic DNA from pure H1-ESCs, and 1/100 and 1/1000 dilutions of 320-GSCs were used as controls.
2. Brightfield images of aging ltGLICO cohort, scale bar = 1cm.
3. Quality control thresholds used for scRNAseq preprocessing.
4. UMAP of the ageing ltGLICO cohort colored by seurat cluster.
5. UMAP of the ageing ltGLI CO cohort colored by sourporcell genotype assignment (left). Barplot showing proportion of each genotype per seurat cluster.
6. Table showing final number and percentages of genotypes in each ltGLICO sample.

**
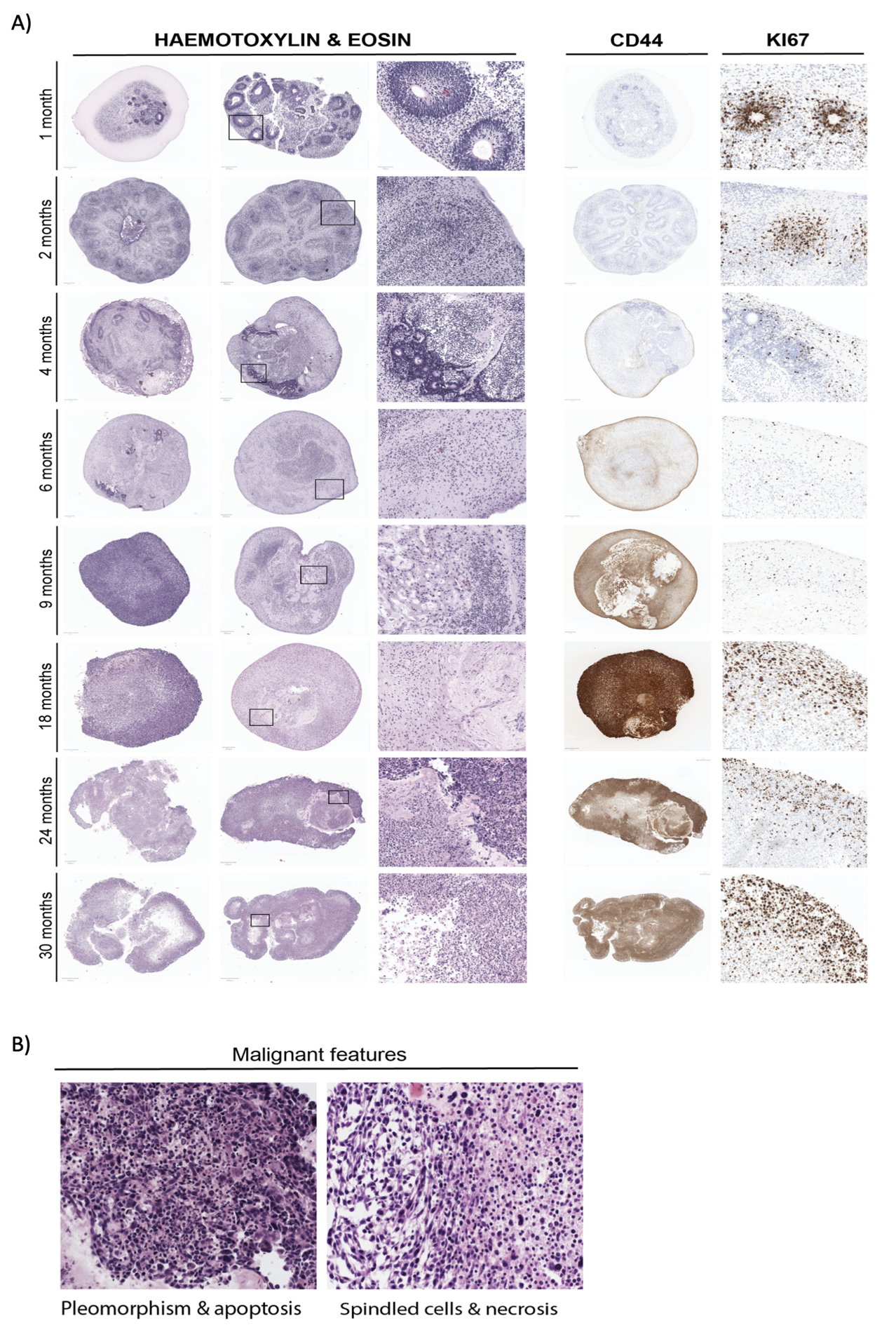
**

**Figure S2: Histopathological analysis of the ltGLICO cohort.**

1. Left, Haemotoxylin and Eosin staining of the aging ltGLICO cohort (1-30months), sections from 2 representative ltGLICOs shown, zoomed inset highlights regions of histological interest. Right, immunohistochemical staining of the aging ltGLICO cohort (1-30months) for CD44 and Ki67.
2. Representative Haemotoxylin and Eosin images of malignant histological features observed in older ltGLICOs.

**
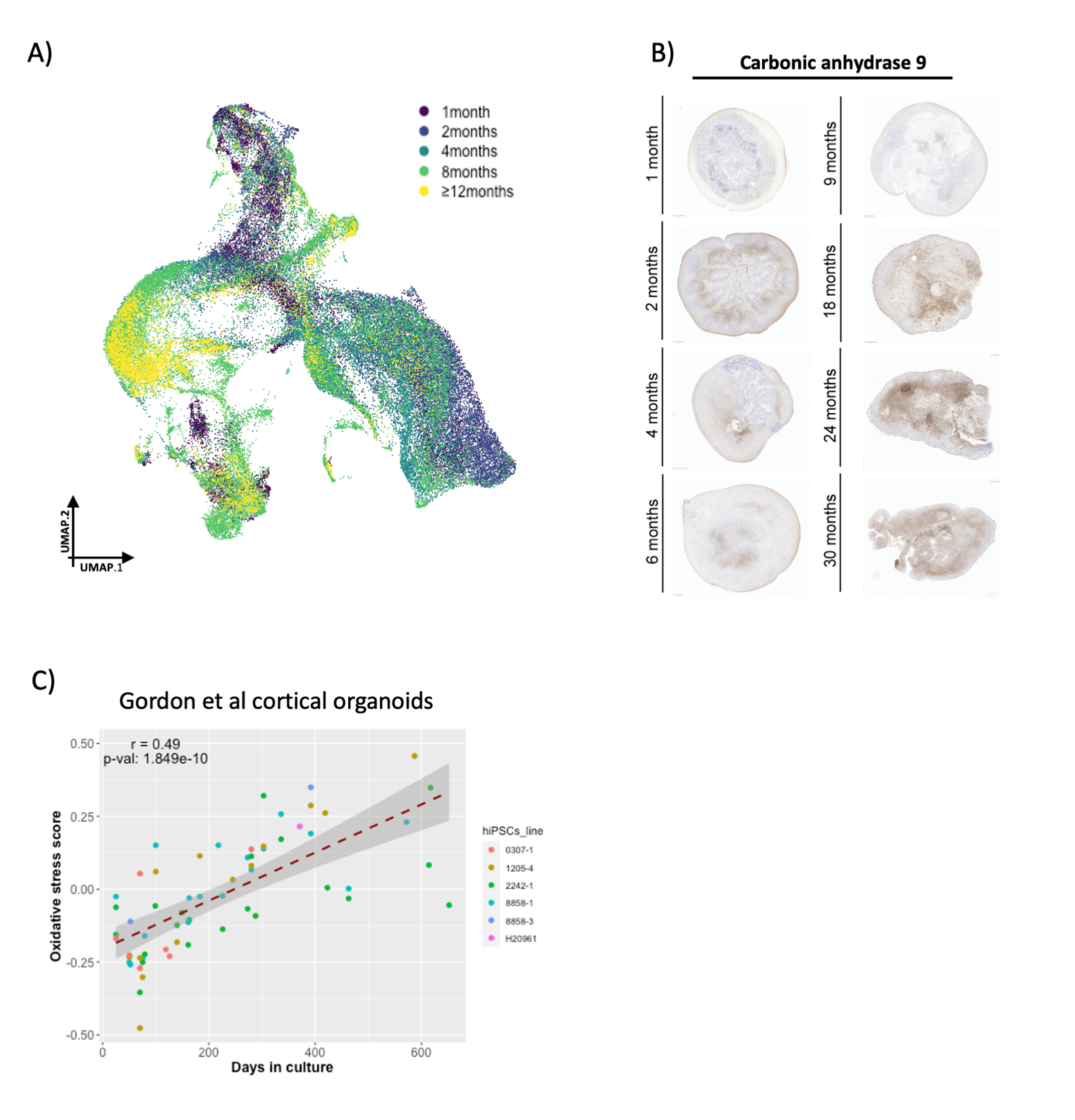
**

**Figure S3 Aging organoids show increased hypoxia and oxidative stress**

1. UMAP of the CO cells from the ageing ltGLICO cohort colored by sample age.
2. Immunohistochemical staining of the aging ltGLICO cohort (1-30months) Carbonic anhydrase 9.
3. Gene set scoring for the Oxidative_stress shows correlation between organoid age and oxidative stress in a reference dataset of 62 RNAseq samples, r=0.49, p < 1.849e-10.


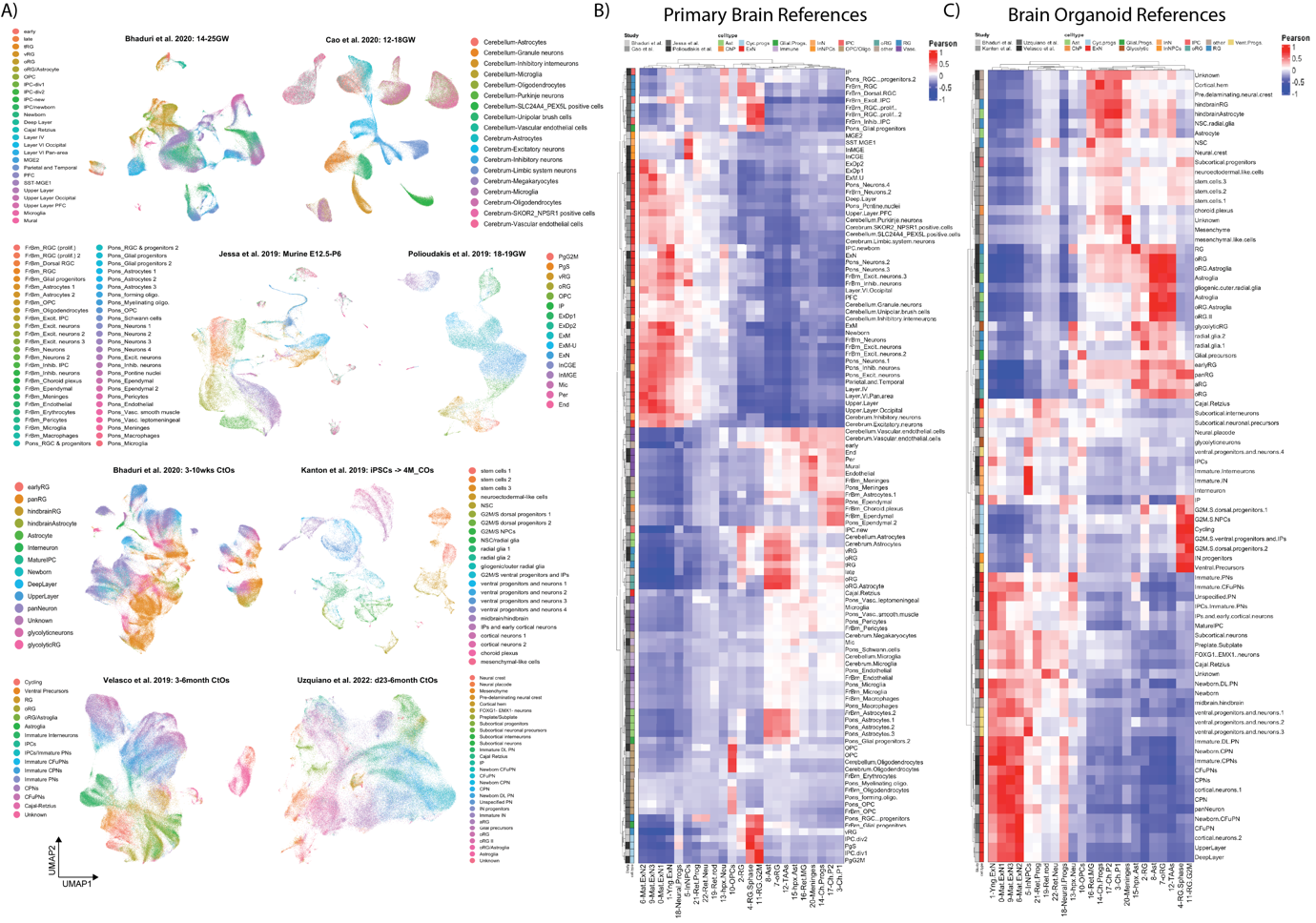


**Figure S4: Correlation of ltGLICO organoid cells to brain organoid and primary fetal brain reference datasets.**

1. UMAP projections of primary developing brain (top; Bhaduri et al, Cao et al, Jessa et al, Polioudakis et al) and brain organoid (bottom; Bhaduri et al, Kanton et al, Velasco et al, Uzquiano et al)reference datasets. All cell type assignments taken from the original publications.
2. Pearson correlation of CO cell subtype marker gene expression with primary brain reference cell type gene expression profiles.
3. Pearson correlation of CO cell subtype marker gene expression with brain organoid reference cell type gene expression profiles.

**
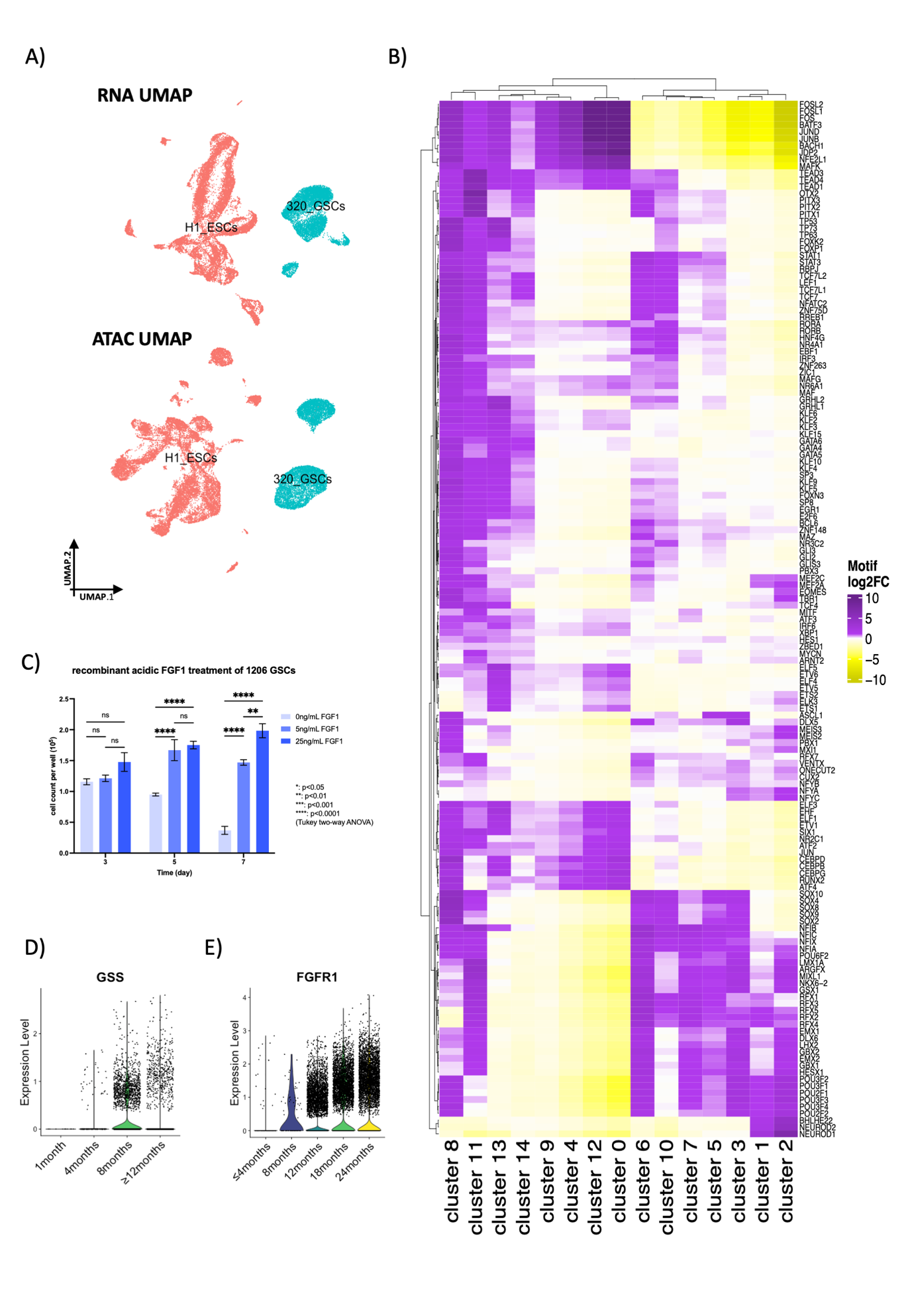
**

**Figure S5: scMulti-ome analysis of 5-12month ltGLICOs**

1. UMAPs of 5-12month ltGLICOs calculated on RNA or ATAC single cell data
2. Cluster specific enrichment of transcription factor motifs, the top 20 regulators per cell type are shown.
3. Effect of recombinant acidic FGF1 on 1206_GSC growth
4. Violin plot of astrocyte glutathione synthetase (GSS) expression in aging ltGLICO cohort, cells grouped by sample age.
5. Violin plot of 320-GSCs FGFR1 expression in aging ltGLICO cohort, cells grouped by sample age.
